# Craniofacial skeletal pattern: is it really correlated with the degree of adenoid obstruction?

**DOI:** 10.1590/2176-9451.20.4.068-075.oar

**Published:** 2015

**Authors:** Murilo Fernando Neuppmann Feres, Tomas Salomão Muniz, Saulo Henrique de Andrade, Maurilo de Mello Lemos, Shirley Shizue Nagata Pignatari

**Affiliations:** 1 Associate professor, Universidade São Francisco, Department of Orthodontics, Bragança Paulista, São Paulo, Brazil; 2 Private practice, Bragança Paulista, São Paulo, Brazil; 3 Full professor, Universidade Guarulhos, Department of Orthodontics, Guarulhos, São Paulo, Brazil; 4 Adjunct professor of Otolaryngology, Universidade Federal de São Paulo, Department of Otolaryngology, Head and Neck Surgery, São Paulo, São Paulo, Brazil

**Keywords:** Mouth breathing, Diagnosis, Angle Class II malocclusion

## Abstract

**OBJECTIVE:**

The aim of this study was to compare the cephalometric pattern of children with and without adenoid obstruction.

**METHODS:**

The sample comprised 100 children aged between four and 14 years old, both males and females, subjected to cephalometric examination for sagittal and vertical skeletal analysis. The sample also underwent nasofiberendoscopic examination intended to objectively assess the degree of adenoid obstruction.

**RESULTS:**

The individuals presented tendencies towards vertical craniofacial growth, convex profile and mandibular retrusion. However, there were no differences between obstructive and non-obstructive patients concerning all cephalometric variables. Correlations between skeletal parameters and the percentage of adenoid obstruction were either low or not significant.

**CONCLUSIONS:**

Results suggest that specific craniofacial patterns, such as Class II and hyperdivergency, might not be associated with adenoid hypertrophy.

## Abstract (pt)

**OBJETIVO:**

a presente pesquisa teve como objetivo comparar o padrão cefalométrico de crianças com e sem obstrução adenoidiana.

**MÉTODOS:**

a amostra consistiu de 100 crianças, com idades entre 4 e 14 anos, de ambos os sexos, submetidas a exames cefalométricos para a avaliação de variáveis cefalométricas horizontais e verticais. A amostra também foi submetida à nasofibroendoscopia, por meio da qual o grau de obstrução adenoidiana foi objetivamente aferido.

**RESULTADOS:**

os pacientes avaliados demonstraram tendência ao crescimento vertical acentuado, ao perfil convexo e à retrusão mandibular. No entanto, não houve diferenças entre pacientes portadores e não portadores de obstrução, em relação a todas as variáveis cefalométricas. As correlações estabelecidas entre os parâmetros esqueléticos e os percentuais de hipertrofia foram baixas ou não significativas.

**CONCLUSÕES:**

os resultados sugerem que padrões faciais específicos, tais como Classe II e hiperdivergência, parecem não estar associados à hipertrofia adenoideana.

## INTRODUCTION

Studies on the relationship between respiratory pattern and the development of craniofacial characteristics have been published for a considerable period of time.^1^^-^ 8 The persistence of the interest in this topic might be partially explained by the high prevalence of mouth breathing,^9^ even among orthodontic patients.^10^

The presence of this habit is correlated with a number of muscular^11^ and dento-craniofacial alterations, including maxillary constriction, posterior crossbite, retrusion and clockwise rotation of the mandible, Class II skeletal pattern and excessive vertical growth.^1^^-^ 8

One of the main causes of mouth breathing is adenoid hypertrophy,^12^ In addition, several studies have demonstrated a significant correlation between long face morphology and anatomical reduction in the nasopharyngeal airway.^1^^,^ 7 ^,^ 8 ^,^ 13 ^,^ 14 ^,^ 16 ^-^ 19 Class II malocclusion or mandibular retrognathia have also been frequently related to smaller dimensions of the nasopharynx.^13^^,^ 20 ^-^ 23 Therefore, some of these studies^7^^,^ 14 ^,^ 16 ^,^ 18 ^,^ 24 suggest that dimensional reduction of the nasopharynx, due to hyperdivergent craniofacial pattern or mandibular retrusion, might predispose patients to an obstructive breathing status derived from adenoid hypertrophy. However, such inferences might be considered mere assumptions rather than scientific evidence, since most of these studies have relied upon inaccurate methods, such as rhinomanometry^1^^,^ 13 ^,^ 21 or lateral cephalometric radiographs,^7^^,^ 8 ^,^ 14 ^,^ 16 ^,^ 17 ^,^ 18 ^,^ 19 ^,^ 20 ^,^ 22 to assess patients' respiratory pattern. On the other hand, nasofiberendoscopic examination has been considered as the gold standard method for adenoid evaluation.^23^

The primary objective of this study was to describe the craniofacial morphology of patients with complaints of nasopharyngeal obstruction. In addition, comparative analysis of cephalometric skeletal features was conducted on patients with and without adenoid hypertrophy, as assessed by nasofiberendoscopic examination. Finally, this study also aimed to investigate the correlations established between skeletal characteristics and the percentage of adenoid obstruction.

## MATERIAL AND METHODS

This research was a descriptive-analytical, cross-sectional study approved by Universidade Federal de São Paulo Institutional Review Board (protocol #0181/08).

Between February 2009 and June 2010, 170 children who attended or were referred to a public pediatric otolaryngology outpatient clinic, were invited to take part in the study, from which 43 refused to participate. The convenience sample thus consisted of 127 individuals, both males and females, aged between four to 14 years old. In order to be eligible to the study, the children should have presented complaints of nasopharyngeal obstruction and/or mouth breathing. At this point, no objective information regarding the degree of adenoid hypertrophy was available.

Children with syndromes or craniofacial malformation, as well as those which had been previously subjected to orthodontic treatment, were not included in the sample.

All eligible participants, along with their parents or legal guardians, were properly informed about the study objectives and procedures, as well as the examinations that would be performed. Those who agreed to participate formalized their intent by signing an informed consent form previously prepared according to Universidade Federal de São Paulo Institutional Review Board.

Initially, the children selected underwent lateral cephalometric radiographic examination performed by a radiology specialist who used the same device for all of them (Instrumentarium Ortopantomographic OP100; General Electric Healthcare, Tuusula, Finland). The focus-film distance was 140 cm, while X-ray exposure settings were 70 kV and 12 mA for 0.40 to 0.64 s. During record taking, patients were instructed to breathe exclusively through the nose, keep their mouth closed and their teeth in occlusion. We used 20 x 25 cm films (Kodak, Rochester, NY) and processed them according to a standardized protocol. Radiographs were identified by codes, so as to prevent patient identification.

**Figure 1.**
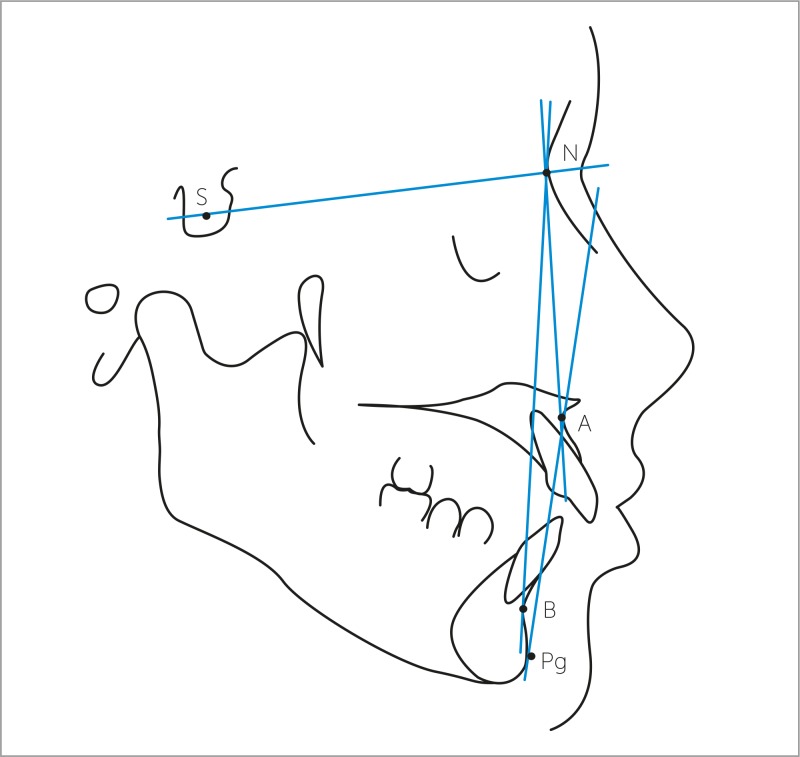
Horizontal cephalometric variables (SNA, SNB, ANB, NAPg).

**Figure 2.**
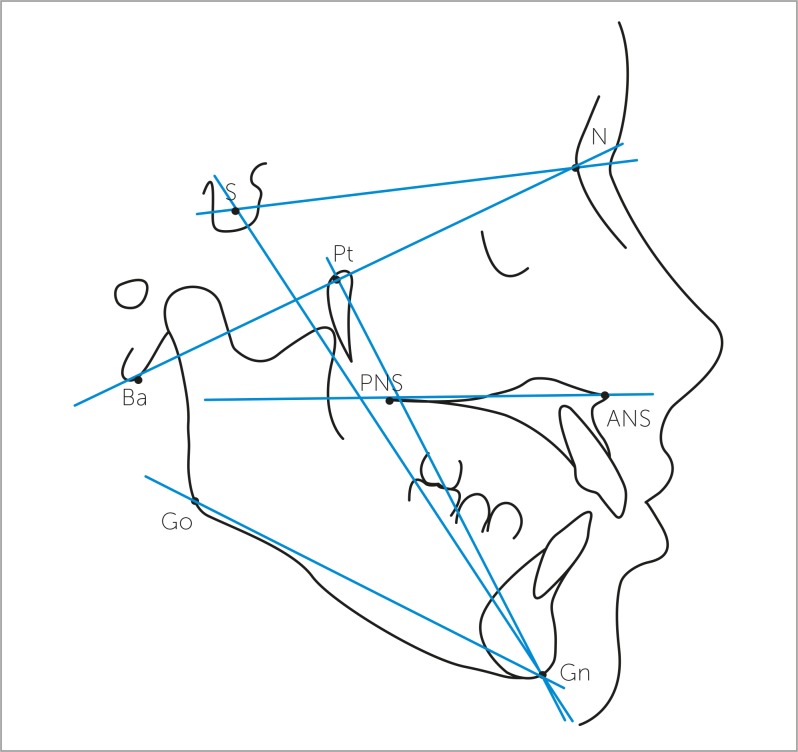
Vertical cephalometric variables (NSGn, SNGoGn, SNPP, BaNPtGn).

**Figure 3.**
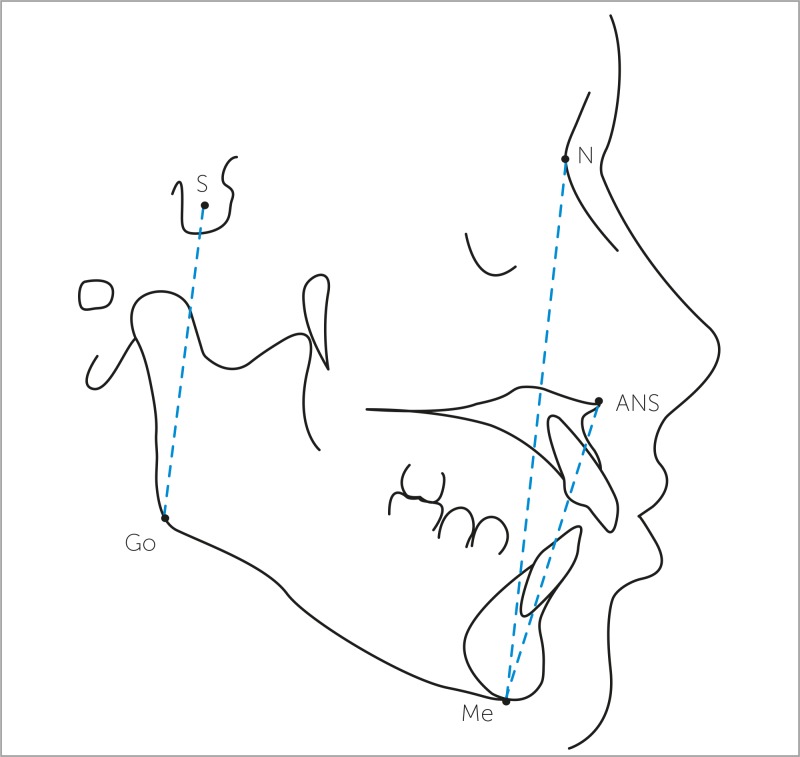
Cephalometric variables related to the facial indices AFHi and FHi.

Lateral cephalometric radiographs were manually traced by two independent blind examiners, and subsequent measurements (Table 1, Figs 1, 2, and 3) were performed on Ultraphan acetate sheets, with the aid of a light box, a protractor and a digital caliper (model 799A-8/200; Starrett , Itu, Brazil), with 0.01 mm precision.

**Table 1.** Cephalometric variables evaluated

Cephalometric variables	
SNA	Anteroposterior position of the maxilla
SNB	Anteroposterior position of the mandible
ANB	Relative anteroposterior position of the maxilla and mandible
NAPg	Facial convexity
NSGn	Direction of facial growth
SNGoGn	Mandibular plane angle
SNPP	Inclination of the palatal plane (ANS/PNS)
BaNPtGn	Facial axis
AFHi	Anterior facial height index (ANS-Me/N-Me)
FHi	Total facial height index (S-Go/N-Me)

Subsequently, patients underwent flexible nasofiberendoscopic examination (model ENFP4, 3.4 mm; Olympus, Melville, NY) through both nostrils. The examination was conducted by experienced otolaryngologists and performed after topical anesthesia application (2% lidocaine). All examinations were recorded by a DVD recorder (model DVD-R150/XAZ; Samsung, Manaus, Brazil), and a digital file derived from the primary video was edited to prevent patient identification. Edited video clips were then forwarded to another experienced and independent otolaryngologist who had not been involved with subjects' enrollment, videonasopharyngoscopic examination performance, record taking or editing.

**Figure 4.**
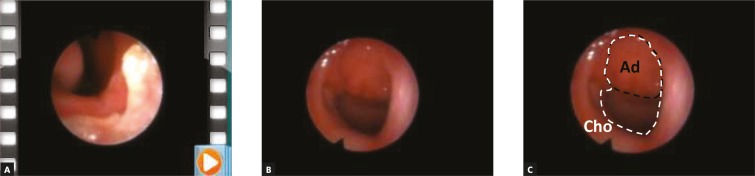
Final frame selection (B) derived from the video clip (A), and MCO calculation (C): MCO = (Ad/Cho) x 100.

In order to evaluate the edited video clips, the examiner used an assessment method originally designed to quantify the degree of obstruction caused by the adenoid tissue: measured choanal obstruction (MCO) which has previously proven to be satisfactorily reproducible.^25^ The evaluator was instructed to choose the frame that provided the best view of the adenoid in relation to the choana, obtained from the most distal portion of the inferior turbinate. In these frames, the patient had to be breathing exclusively through the nose, with no evidence of soft palate elevation. The selected frame was then converted into a JPEG file and the MCO was calculated by means of Image J,^26^ an image processing software. MCO represented the percentage of the choanal area occupied by adenoid tissue (Fig 4). When images from both nostrils were available, the mean between right and left side evaluations was calculated to minimize potential variations, as proposed by Feres et al.^25^

## Statistical analysis

At first, radiographic parameters reliability was determined after intra and inter-reproducibility analysis calculated by intraclass correlation coefficient (ICC).

Then, radiographic variables were described (means, standard deviations, minimum and maximum values) for all subjects. Subsequently, the sample was divided into two groups, according to the degree of obstruction caused by adenoid hypertrophy. According to previous parameters,^27^ patients with MCO ≥ 66.7% were considered to have pathological adenoid hypertrophy, herein denominated the "positive" group; while patients with MCO < 66.7% were not considered to present pathological adenoid hypertrophy ("negative" group). Both groups were compared regarding all cephalometric variables, according to Mann-Withney test.

Finally, Pearson correlation analysis between cephalometric measurements and MCO was determined for the whole sample. The "strength" of the correlation was characterized according to Vieira^28^ as: "irrelevant" (0.00 < r ≤ 0.25), "weak" (0.25 < r ≤ 0.50), "moderate" (0.50 < r ≤ 0.75), or "strong" (0.75 < r ≤ 1.00).

Significance level for statistical tests was set at 5% (α ≤ 0.05). All analyzes were performed using SPSS 10.0 for Windows.

## RESULTS

From the initial sample comprising 127 patients who met the eligibility criteria and agreed to participate in the study, seven were excluded due to inadequate exams and 20 presented inconsistent repeated radiographic measurements. For the 100 remaining patients, all of them had radiographic parameters with satisfactory reproducibility (Table 2).

**Table 2.** Variables Intraexaminer p value Inter-examiner p value SNA (degrees) 0.942 < 0.001 0.950 < 0.001

Variables	Intraexaminer	*p* value	Inter-examiner	*p* value
SNA (degrees)	0.942	< 0.001	0.950	< 0.001
SNB (degrees)	0.966	< 0.001	0.970	< 0.001
ANB (degrees)	0.928	< 0.001	0.913	< 0.001
NAPog (degrees)	0.940	< 0.001	0.926	< 0.001
NSGn (degrees)	0.961	< 0.001	0.945	< 0.001
SnGoGN (degrees)	0.911	< 0.001	0.908	< 0.001
SNPP (degrees)	0.923	< 0.001	0.910	< 0.001
BaNPtGn (degrees)	0.988	< 0.001	0.975	< 0.001
AFHi	0.890	< 0.001	0.877	< 0.001
FHi	0.869	< 0.001	0.868	< 0.001

The final sample comprised 48 female children (48.0%) and 52 male children (52.0%), with a mean age of 9.0 years old (standard deviation = 2.4). The descriptive analysis of all cephalometric variables is presented in Table 3. According to universally accepted parameters, the sample of this study showed skeletal patterns with a slight tendency towards excessive vertical growth, a convex profile and mandibular retrusion. However, comparison between the cephalometric variables of the positive (n = 58, 58.0%) and negative groups (n = 42, 42.0%) showed no statistically significant differences (Table 4).

Furthermore, amongst all radiographic variables evaluated herein, SNA, SNB, NSGn, SNGoGn and BaNPtGn were significantly correlated with MCO. Although statistically significant, the magnitude of these correlations was considered either irrelevant (SNA, SNB, SNGoGn, BaNPtGn) or weak (NSGn) (Table 4).

**Table 3.** Descriptive analysis of radiographic variables.

Variables	Mean	Standard deviation	Minimum	Maximum
SNA (degrees)	82.910	4.3376	70.0	97.5
SNB (degrees)	78.195	3.7281	70.0	93.0
ANB (degrees)	4.715	2.6412	-6.0	11.5
NAPog (degrees)	10.050	5.4802	-8.0	24.0
NSGn (degrees)	69.300	3.9222	56.0	79.0
SnGoGN (degrees)	37.679	5.2047	19.0	48.0
SNPP (degrees)	6.975	3.7902	-4.0	16.0
BaNPtGn (degrees)	87.470	4.1261	78.0	101.0
AFHi	0.588	0.026	0.528	0.705
FHi	0.612	0.041	0.517	0.784

**Table 4.** Comparative analysis between positive (MCO = 66.7%) and negative groups (MCO < 66.7%) in relation to the radiographic variables.

Variables	Groups	Mean	Standard deviation	Mann-Withney (*p* value)
SNA (degrees)	Positive	82.129	4.0410	0.058
	Negative	83.988	4.5472	
SNB (degrees)	Positive	77.612	3.1247	0.290
	Negative	79.000	4.3407	
ANB (degrees)	Positive	4.517	2.9289	0.296
	Negative	4.988	2.1878	
NAPog (degrees)	Positive	9.276	5.8115	0.150
	Negative	11.119	4.8525	
NSGn (degrees)	Positive	69.957	3.8824	0.105
	Negative	68.393	3.8390	
SnGoGN (degrees)	Positive	38.257	4.5389	0.419
	Negative	36.881	5.9703	
SNPP (degrees)	Positive	6.724	3.6685	0.241
	Negative	7.321	3.9707	
BaNPtGn (degrees)	Positive	86.905	4.0819	0.324
	Negative	88.250	4.1072	
AFHi	Positive	0.591	0.027	0.157
	Negative	0.582	0.023	
FHi	Positive	0.611	0.030	0.772
	Negative	0.613	0.054	

**Table 5.** Correlation coefficient (r) between radiographic variables and MCO

Variables	r	Spearman (*p* value)
SNA	-0.250	0.012
SNB	-0.202	0.044
ANB	-0.052	0.605
NAPog	-0.078	0.443
NSGn	0.304	0.002
SNGoGn	0.233	0.020
SNPP	-0.014	0.888
BaNPtGn	-0.242	0.015
AFHi	0.183	0.069
FHi	-0.105	0.298

## DISCUSSION

The association between specific skeletal patterns and the presence of obstructive adenoid is a topic which has been debated for years,^1^^,^ 7 ^,^ 8 ^,^ 13 ^-^ 19 although controversy still remains. One of the reasons that might contribute for this debate to persist is related to the varied sorts of assessment methods used to evaluate the level of adenoid obstruction.

This study has demonstrated that children with respiratory complaints might present skeletal features associated with hyperdivergency and retrognathia. However, despite currently accepted hypotheses according to which dolichofacial or Class II patients are more anatomically susceptible to present adenoid obstruction, evidence presented herein suggests that children are likely to experience it regardless of their skeletal characteristics.

According to most studies, the size of the nasopharyngeal airway is significantly correlated with excessively vertical cephalometric features. Researchers have suggested that this dimensional reduction of the nasopharynx might be attributed to skeletal characteristics which are inherent to hyperdivergent patients, such as maxillomandibular retrusion.^7^^,^ 14 ^,^ 16 ^,^ 18 ^,^ 23 Nevertheless, Santos-Pinto et al^16^ refuted this hypothesis when they demonstrated that individuals with varying nasopharyngeal dimensions did not significantly differ in relation to the anteroposterior position of the maxilla and the mandible. The data obtained in our study support their findings,^16^ since the anteroposterior position of the maxilla and the mandible showed no relevant correlation with the degree of adenoid obstruction, as determined by flexible nasofiberendoscopic examination. Moreover, according to our results, the subjects who were considered to be positive presented similar maxillomandibular sagittal position as those considered to be negative for adenoid obstruction.

That finding might explain why no significant differences were found in relation to ANB when positive and negative groups were compared. In addition, ANB revealed no relevant correlation with the degree of adenoid obstruction. These findings corroborate the results of Freitas et al,^17^ according to which sagittal malocclusions are not correlated with nasopharyngeal airway depth.

Further evidence provided by this study contradicts what other studies^7^^,^ 20 ^,^ 23 ^,^ 25 claim. Some of these researches,^7^^,^ 23 after lateral cephalometric analysis, reported that Class II patients had significantly smaller airway areas. In their latest study on tomographic measurements, Claudino et al^28^ were unable to detect a significant association between nasopharyngeal dimensions and the sagittal skeletal pattern in adolescents. The authors demonstrated that more obvious influence of the skeletal pattern could be observed in relatively lower portions of the pharynx, such as the oropharynx, rather than at the nasopharyngeal level.^28^

Similarly to Claudino et al,^29^ this study found no significant differences between participants with distinct grades of adenoid obstruction, whether vertical or sagittal skeletal parameters. Likewise, no relevant correlations were observed between the percentage of adenoid obstruction and any of the skeletal variables investigated. It is our opinion that most of the studies that have been carried out to date^7^^,^ 8 ^,^ 13 ^,^ 14 ^,^ 16 ^-^ 22 ^,^ 24 have actually failed to infer that patients with specific skeletal patterns (dolichofacial and/or Class II) significantly present higher frequencies of pathological adenoid obstruction. Considering the data obtained herein, it no longer seems reasonable to assume that a reduction in the nasopharyngeal airway is directly related to an actual clinical obstruction. Thüer et al^13^have already reported that there is no significant correlation between nasal airflow parameters, derived from rhinomanometry, and the nasopharyngeal space observed in lateral cephalometric radiograph. The absence of a significant correlation between respiratory capacity and anatomical traits of dolichocephaly has been also reported by Solow et al^21^ who sought to correlate skeletal morphological patterns with data obtained from rhinomanometry examination.

In addition, although imaging techniques may indeed indicate nasopharyngeal anatomical reduction, these might not be able to promote significant influence on patient's clinical respiratory conditions, nor necessarily predispose one to effectively develop obstruction. Unlike many other researches, in this study, a direct and visual nasopharyngeal evaluation method was used, which, according to relevant literature,^23^ is considered to be the gold standard for adenoid evaluation.

However, this study presents significant limitations, with the most important one being associated with single cross-sectional evaluation of adenoid hypertrophy. As previously reported,^31^ the adenoid lymphoid tissue might be susceptible to sudden dimensional changes as a consequence of allergic sensitization. Therefore, the authors suggest that future studies should address this limitation by performing serial adenoid evaluations, so as to minimize potential variations. In addition, new research is still required to investigate the influence of other morphological parameters, such as those related to the cranial base,^30^ on the dimensional reduction of the nasopharynx and the potential establishment of an obstructive respiratory process, since this study was limited to assess only maxillary or mandibular parameters.

## CONCLUSION

The sample studied herein showed skeletal patterns with a discrete tendency towards excessive vertical growth, a convex profile and mandibular retrusion. However, no statistically significant differences were found between patients with or without adenoid hypertrophy. The correlations established between the characteristics of craniofacial morphology and the percentage of choanal obstruction were weak or not significant.

## References

[1] Linder-Aronson S (1970). Their effect on mode of breathing and nasal airflow and their
relationship to characteristics of the facial skeleton and the dentition. A
biometric, rhino-manometric and cephalometro-radiographic study on children with
and without adenoids. Acta Otolaryngol Suppl.

[2] Melsen B, Attina L, Santuari M, Attina A (1987). Relationships between swallowing pattern, mode of respiration, and
development of malocclusion. Angle Orthod.

[3] Löfstrand-Tideström B, Thilander B, Ahlqvist-Rastad J, Jakobsson O, Hultcrantz E (1999). Breathing obstruction in relation to craniofacial and dental arch
morphology in 4-year-old children. Eur J Orthod.

[4] Sabatoski CV, Maruo H, Camargo ES, Oliveira JHG (2002). Estudo comparativo de dimensões craniofaciais verticais e horizontais
entre crianças respiradoras bucais e nasais. J Bras Ortodon Ortop Facial.

[5] Lopatiene K, Babarskas A (2002). Malocclusion and upper airway obstruction. Medicina (Kaunas).

[6] Lessa FC, Enoki C, Feres MF, Valera FC, Lima WT, Matsumoto MA, Breathing mode influence in craniofacial development (2005). Braz. J Otorhinolaryngol.

[7] Wysocki J, Krasny M, Skarzynski PH (2009). Patency of nasopharynx and a cephalometric image in the children with
orthodontic problems. Int J Pediatr Otorhinolaryngol.

[8] Ucar FI, Uysal T Orofacial airway dimensions in subjects with Class I malocclusion
and different growth patterns (2011). Angle. Orthod.

[9] de Menezes VA, Leal RB, Pessoa RS, Pontes RM (2006). Prevalence and factors related to mouth breathing in school children
at the Santo Amaro project-Recife, 2005. Braz J Otorhinolaryngol.

[10] di Francesco RC, Bregola EGP, Pereira LS, Lima RS (2006). A obstrução nasal e o diagnóstico ortodôntico. Rev Dent Press Ortod Ortop Facial.

[11] Valera FC, Travitzki LV, Mattar SE, Matsumoto MA, Elias AM, Anselmo-Lima WT (2003). Muscular, functional and orthodontic changes in pre school children
with enlarged adenoids and tonsils. Int J Pediatr Otorhinolaryngol.

[12] Farid M, Metwalli N (2010). Computed tomographic evaluation of mouth breathers among paediatric
patients. Dentomaxillofac Radiol.

[13] Thüer U, Kuster R, Ingervall B (1989). A comparison between anamnestic, rhinomanometric and radiological
methods of diagnosing mouth-breathing. Eur J Orthod.

[14] Joseph AA, Elbaum J, Cisneros GJ, Eisig SB (1998). A cephalometric comparative study of the soft tissue airway dimensions
in persons with hyperdivergent and normodivergent facial patterns. J Oral Maxillofac Surg.

[15] Akcam MO, Toygar TU, Wada T (2002). Longitudinal investigation of soft palate and nasopharyngeal airway
relations in different rotation types. Angle Orthod.

[16] Santos-Pinto A, Paulin RF, Melo ACM, Martins LP (2004). A influência da redução do espaço nasofaringeano na morfologia facial
de pré-adolescentes. Rev Dental Press Ortod Ortop Facial.

[17] Freitas MR, Alcazar NM, Janson G, Freitas KM, Henriques JF (2006). Upper and lower pharyngeal airways in subjects with Class I and Class
II malocclusions and different growth patterns. Am J Orthod Dentofacial Orthop.

[18] Feres MFN, Enoki C, Anselmo-Lima WT, Matsumoto MAN (2010). Dimensões nasofaringeanas e faciais em diferentes padrões
morfológicos. Dental Press J Orthod.

[19] Macari AT, Bitar MA, Ghafari JG (2012). New insights on age-related association between nasopharyngeal airway
clearance and facial morphology. Orthod Orthod Craniofac Res.

[20] Mergen DC, Jacobs RM (1970). The size of nasopharynx associated with normal occlusion and Class II
malocclusion. Angle Orthod.

[21] Solow B, Siersbaek-Nielsen S, Greve E (1984). Airway adequacy, head posture, and craniofacial
morphology. Am J Orthod.

[22] Krasny M, Wysocki J, Zadurska M, Skarzynski PH (2011). Relative nasopharyngeal patency index as possible objective indication
for adenoidectomy in children with orthodontic problems. Int J Pediatr Otorhinolaryngol.

[23] Mlynarek A, Tewfik MA, Hagr A, Manoukian JJ, Schloss MD, Tewfik TL (2004). Lateral neck radiography versus direct video rhinoscopy in assessing
adenoid size. J Otolaryngol.

[24] Alves M, Franzotti ES, Baratieri C, Nunes LK, Nojima LI, Ruellas AC (2012). Evaluation of pharyngeal airway space amongst different skeletal
patterns. Int J Oral Maxillofac Surg.

[25] Feres MF, Hermann JS, Sallum AC, Pignatari SS (2013). Endoscopic evaluation of adenoids: reproducibility analysis of current
methods. Clin Exp Otorhinolaryngol.

[26] ImageJ (1997). US National Institutes of Health.

[27] Chien CY, Chen AM, Hwang CF, Su CY (2005). The clinical significance of adenoid-choanae area ratio in children
with adenoid hypertrophy. Int J Pediatr Otorhinolaryngol.

[28] Vieira S (2008). Introdução à Bioestatística.

[29] Claudino LV, Mattos CT, Ruellas AC, Sant' Anna EF (2013). Pharyngeal airway characterization in adolescents related to facial
skeletal pattern: a preliminary study. Am J Orthod Dentofacial Orthop.

[30] Martin O, Muelas L, Viñas MJ (2011). Comparative study of nasopharyngeal soft-tissue characteristics in
patients with Class III malocclusion. Am J Orthod Dentofacial Orthop.

[31] Modrzynski M, Zawisza E (2007). The influence of birch pollination on the adenoid size in children
with intermittent allergic rhinitis. Int J Pediatr Otorhinolaryngol.

